# Interaction of Wheat Bran Particle Size and Stimbiotic Supplementation on Growth Performance and Gut Health Parameters in Broilers

**DOI:** 10.3390/ani14182685

**Published:** 2024-09-15

**Authors:** Shravani Veluri, Mike R. Bedford, Gemma Gonzalez-Ortiz, Oluyinka Abiona Olukosi

**Affiliations:** 1 Department of Poultry Science, University of Georgia, Athens, GA 30602, USA; oaolukosi@uga.edu; 2 AB Vista, Marlborough, Wiltshire SN8 4AN, UK; mike.bedford@abvista.com (M.R.B.); gemma.gonzalez@abvista.com (G.G.-O.)

**Keywords:** stimbiotic, wheat bran, particle size, short-chain fatty acids, ileal nutrient digestibility

## Abstract

**Simple Summary:**

Wheat bran inclusion beneficially affects the gut health of broilers at low inclusion levels, along with the supplementation of feed additives such as with a stimbiotic It was hypothesized that the coarse or fine particle size of the wheat bran has a significant influence and interaction with stimbiotic on the performance of, and gut-health beneficial effects, in broilers. Coarse- or fine-wheat-bran inclusion or stimbiotic inclusion increased the feed intake and FCR for younger broilers; however, fine wheat bran and stimbiotic inclusion increased the overall weight gain of broilers without influencing their feed intake and FCR. Fine-wheat-bran inclusion increased the jejunum villi height and ileal nutrient digestibility compared to coarse wheat bran or diets without wheat bran in broilers at day 18. Stimbiotic supplementation increased ileal nutrient digestibility at day 42. The particle size of the wheat bran or stimbiotic supplementation had no effects on the cecal total short-chain fatty acid concentration; however, stimbiotic supplementation or wheat bran inclusion tended to decrease the branched-chain fatty acid concentration. Stimbiotic supplementation and inclusion of wheat bran with a reduced particle size into broiler diets have beneficial effects on the overall performance and ileal nutrient digestibility.

**Abstract:**

A 42-day study was conducted with 720-day-old Cobb male broiler chicks allocated to treatments in a 3 × 2 factorial, with the factors as wheat bran (WB) inclusion (no WB, 50 g/kg coarse WB, or 50 g/kg fine WB) and stimbiotic (STB) supplementation in corn-based diets. The inclusion of WB (*p* < 0.05) or STB supplementation (*p* < 0.05) increased the FCR and feed intake in the day 0–10 phase. During the day 0–28 phase, coarse-WB inclusion increased (*p* < 0.05) the FCR, compared to fine WB or diets without WB. In the day 0–42 phase, WB marginally decreased weight gain in diets without STB supplementation, but the STB-supplemented diet, weight gain was greater (*p* < 0.05) the diet with fine WB compared with diets with coarse WB. Fine-WB inclusion increased the ileal nitrogen and energy digestibility determined at day 18 compared to coarse WB or diets without WB. Supplementation with STB (*p* < 0.05) or fine WB (*p* < 0.05) inclusion increased the villi height compared to diets without STB supplementation or coarse WB, or the diet without WB. Coarse or fine WB decreased (*p* < 0.05) cecal branched-chain fatty acids compared to diets without WB. In conclusion, stimbiotic supplementation to fine WB improved the performance and nutrient digestibility of broilers compared to coarse WB with no effects on the caeca total SCFA concentration.

## 1. Introduction

Supplementing broiler diets with xylanase and xylooligosaccharides (XOS) yields positive effects through a stimbiotic mechanism, a term recently introduced to describe feed additives that enhance the activity of fiber-fermenting microbiota in the ceca without significantly contributing to cecal short-chain fatty acid (SCFA) production [1]. Xylanase and XOS, integral components of stimbiotics, work synergistically: xylanase breaks down soluble arabinoxylan in the feed into insoluble arabinoxylo-oligosaccharides for fermentation in the ceca, promoting the development of a fiber-fermenting microbiome. The fiber fermentation capacity of young broiler chickens is naturally limited due to a deficiency in enzymes required for fiber hydrolysis [2]. Adding extra XOS stimulates the fiber-fermenting microbiome in the ceca, fostering the establishment of a microbiome with enhanced fiber-fermenting capabilities. This microbiome accelerates the fermentation of the soluble arabinoxylans, the amount of which is increased through xylanase use. The prebiotic nature of XOS is well documented, and selectively utilized by lactate and butyrate-producing bacteria, with lactate serving as a cross-feeding substrate for butyrate-producing bacteria [3]. An increase in fiber fermentation increases the production of SCFAs and bacterial xylanase and cellulases, which can further act on dietary fiber in the digesta. Consequently, arabinoxylo-oligosaccharides released from arabinoxylan by xylanase can be utilized by the established fiber-fermenting microbes to produce SCFA as fermentation end-products. This increase in oligosaccharide and SCFA production offers multiple benefits, including the maintenance of gut integrity and immunity, and promoting overall gut health.

Corn-based diets have lower arabinoxylan and fiber contents for xylanase to act on and increase fiber fermentation. The inclusion of additional fiber sources like wheat bran increases the substrates for stimbiotics to act on. Wheat bran is a source of insoluble dietary fiber and is rich in arabinoxylans. The arabinoxylan content of wheat bran is estimated to be around 23.2% [4]. Wheat bran, when included at appropriate levels in broiler diets, promotes gizzard development and the secretion of endogenous enzymes [5]. This altogether increases the nutrient digestibility and performance of broilers [6]. Other beneficial effects of wheat bran include immune modulatory effects and anti-oxidative capacity, which comes from the free-radical-scavenging activity of the phenolic compounds present, tocopherol and carotenoid [7,8]. Wheat bran, when included along with stimbiotic supplementation into corn-based diets, was hypothesized to improve the performance of broilers through an increase in the fermentation capacity of the ceca.

Wheat bran particle size has also been shown to influence the fermentation capacity in the ceca of broilers [9]. A coarse particle size of the wheat bran was shown to stimulate gizzard development and increase the digestibility of nutrients by stimulating the release of cholecystokinin, which increases the release of digestive enzymes. A fine particle size of the wheat bran has a higher fermentation capacity in the ceca because its reduced particle size allows it to enter the ceca easily to undergo fermentation [9]. Reduced- or fine-particle-size wheat bran also has a higher antioxidant capacity compared to the coarse size [10] and also increases the surface area for xylanase or exogenous enzymes to act on [11]. The hypothesis of this experiment is that the fine particle size of the wheat bran increases the fermentation capacity in the ceca compared to a coarse particle size when supplemented with stimbiotics in corn-based diets. An increase in fermentation capacity leads to an increase in the total SCFA content, whose beneficial effects include improvements in the performance of broiler chickens through an increase in nutrient digestibility and jejunum histomorphology. The objective of the current study was to determine and compare the effects of coarse- or fine-wheat-bran inclusion with or without stimbiotic supplementation into broiler diets on growth performance, ileal nutrient digestibility and oligosaccharide concentration, jejunum morphometrics, and cecal SCFA concentration.

## 2. Materials and Methods

### 2.1. Experimental Design

The study was approved by the Institutional Animal Care and Use Committee at the University of Georgia (Athens, GA, USA; IACUC number: A2021-06-006). The 720 zero-day-old male broiler chicks (male by-products of the female line) from a commercial breeder were allocated to 48 floor pens with 15 birds/pen. There were 6 treatments arranged in a 3 × 2 factorial arrangement with 8 replicates/ treatment. Factors included wheat bran inclusion (0 or 50 g/kg coarse, or 50 g/kg fine wheat bran) and stimbiotic supplementation (Signis, β-1,4-endo-xylanase, and xylo-oligosaccharides, AB Vista, Marlborough, UK) inclusion (0 or 100 g/ton of feed). The study lasted for 42 days, and diets were fed in three phases as starter (days 0 to 10), grower (days 10 to 28), and finisher (days 28 to 42) phases. The diet formulas are presented in Table 1, Table 2 and Table 3, respectively. 

Titanium dioxide was added at 0.3% into the diets only in the grower and finisher phases for nutrient digestibility purposes. Ad libitum access to feed and water was given to the birds, and temperature and lighting schedules were followed according to the Cobb Broilers management guide (2021) [12] Phytase and xylanase activity (in the stimbiotic supplementation) in the diet samples were determined using ELISA (AB Vista, Plantation, FL, USA) and are presented in Table 4.

Body weight and feed intake data were collected on day 10, day 28, and day 42 to determine weight gain, feed intake, and the mortality-corrected FCR. On day 18 and day 42, gizzard, jejunum, ileum, and cecal contents were collected and placed on dry ice, and later transferred to a freezer at –20 °C pending further analysis. The mid-section of the jejunum tissue was collected and part of it was placed in 10% neutral buffered formalin for histomorphology purposes, and another was snap frozen in liquid nitrogen for mRNA expression analysis and later transferred to a freezer at –80 °C for storage. 

### 2.2. Particle Size Analysis of Wheat Bran

The particle size analysis of WB was carried out in the Poultry Science Department at the North Carolina State University and the procedure followed was according to the protocol described in [13] using a 15-sieve stack and a Ro-Tap shaker (Model RX-29 W. S. Tyler’s Ro-Tap®, Mentor, OH). Calculations of geometric mean particle size and the geometric standard deviation of particle diameter by mass were carried out using the ASABE method S319.4 [14]. The geometric mean diameters (GMDs) of the coarse and fine WB used for this study were 1044 µm and 460 µm, respectively. 

### 2.3. Chemical Analyses

#### 2.3.1. Digestibility

Ileal digesta samples were freeze-dried using (Labconco Freeze Dryer: Console Freeze Dryer, Model: 710621115) and then ground using a 0.5 mm sieve (Retsch ZM 200, Retsch GmbH and Co., KG, Germany). To determine the dry matter, 1 g of the diets and 0.6 g of the digesta were dried in a drying oven at 100 °C for 24 h (VWR International Radnor, PA) and the weight difference was noted to determine the dry matter content of the sample using the AOAC method (Method 934.01). The concentration of the external indicator TiO_2_ was determined following the method of Short et al. [15] with modifications as described by Veluri and Olukosi [16]. The gross energy of the diets and digesta in 1 g of a sample was determined using an isoperibol bomb calorimeter (Model 6200, Parr Instruments, Moline, IL, USA) using benzoic acid as a calibration standard, and each sample was run in duplicate. The nitrogen contents of the samples were determined using a LECO FP 828-MC nitrogen analyzer (Method 968.06).

Ileal nutrient digestibility calculations:

(1) $$\mathrm{DMD}\;(\%)=\left({DM}_{diet}-\left[{DM}_{ileal\;digesta}\times \left(\frac{C_{diet}}{C_{ileal\;digest}}\right)\right]\right)\times 100$$

(2) $$\mathrm{IDE}\;(\mathrm{MJ}/\mathrm{kg})=\left({GE}_{diet}-\left[{GE}_{ileal\;digesta}\times \left(\frac{C_{diet}}{C_{ileal\;digest}}\right)\right]\right)$$

(3) $$\mathrm{IND}\;(\%)=\left(N_{diet}-\left[N_{ileal\;digesta}\times \left(\frac{C_{diet}}{C_{ileal\;digest}}\right)\right]\right)\times 100$$

where

DMD = dry matter digestibility

DM*_diet_* = dry matter content in the diet

DM*_ileal digesta_* = dry matter content in the ileal digesta

C*_diet_* = TiO_2_ content in the diet

C*_ileal digesta_* = TiO_2_ content in the ileal digesta

IDE = ileal digestible energy

GE*_diet_* = gross energy content in the diet

G*_ileal digesta_* = gross energy content in the ileal digesta

IND = ileal nitrogen digestibility

N*_diet_* = nitrogen content in the diet

N*_ileal digesta_* = nitrogen content in the ileal digesta

#### 2.3.2. The pH of Gizzard, Jejunum, and Cecal Contents

Gizzard, jejunum, and cecal contents were thawed after removing from the freezer at −20 °C to determine the pH. Samples were weighed out and diluted with water in a 1:9 ratio and later stirred with a magnetic stirrer for 5 min for uniform distribution. Then, a pH probe was placed into the solution and the reading was determined with a digital analog pH meter (Thermofisher Scientific, Waltham, MA, USA). 

#### 2.3.3. Jejunum Histomorphology

Jejunum tissue samples preserved in 10% neutral buffered formalin underwent dehydration using varying alcohol concentrations of 70%, 80%, and 95%. Subsequently, alcohol-cleared samples were embedded in paraffin wax, and the subsequent procedures were carried out following the protocol outlined by Olukosi [17]. Thinly sliced wax blocks were then transferred into a hot water bath and then into the slide. All the slides were stained with hematoxylin and eosin and viewed under a light microscope with 4X magnification (BZ-X800, Keyence Inc., Itasca, IL), and images were captured with a Leica DC500 camera. Using NIH Image J software, a minimum of five random measurements for villi height (VH), crypt depth, and villi width (VW) were taken for each sample. 

#### 2.3.4. Real-Time PCR Analysis

For mRNA expression analysis, RNA was extracted from the jejunum tissue using a QIAzol lysis reagent. Approximately 0.1 g of the jejunum tissue samples were homogenized in QIAzol lysis reagent using a bead beater. After homogenization, 200 µL of chloroform was added and centrifuged at 12,000× *g* for 15 min. The top layer with nucleic acids was transferred to another tube, and to this, one volume of isopropanol was added and placed on ice for 10 min and centrifuged again at 12,000× *g* for 10 min to obtain the RNA pellet at the bottom of the tube. Later, the RNA pellet was washed with ethanol two to three times, and then, the RNA pellet was dissolved in 50–100 μL of nuclease-free water. Using a Nanodrop 2000 spectrophotometer, the quantity and quality of the RNA were determined. Reverse transcription of the RNA into cDNA was performed using a high-capacity cDNA reverse-transcription kit (Applied Biosystems, Life Technologies, Carlsbad, CA, USA). The cDNA samples were run in duplicate for real-time PCR analysis using the iTAQ SYBR green master mix (Bio-Rad, Hercules, CA, USA). The aβ-actin gene was used as housekeeping gene for normalization across the samples and a fold change was calculated using the formula $2^{-\Delta \Delta Ct}$ [18]. The primer sequence of the housekeeping gene, glucose, and protein/amino acid transporters, and tight junction genes are presented in Table 5.

#### 2.3.5. Cecal Short-Chain-Fatty-Acid Analysis

Thawed cecal contents were mixed with distilled water in a 1:3 ratio and centrifuged to clear the dense particles. To the supernatant, freshly prepared metaphosphoric acid was added in a 1:5 ratio and subsequently frozen overnight to facilitate protein precipitation. The next day, the samples were thawed and centrifuged, and ethyl acetate was added to the supernatant to extract short-chain fatty acids into the top layer, and this was later transferred to a screw-thread glass vial for gas chromatography (Shimadzu GC-2010 plus; Shimadzu Corporation, Kyoto, Japan) as described by Lourenco et al. [19].

#### 2.3.6. Oligosaccharide Analysis

Ileal digesta oligosaccharide analysis was conducted using matrix-assisted laser desorption–ionization mass spectrometry, following a protocol adapted from Lin and Olukosi [20]. Freeze-dried and finely ground ileal digesta samples (30 mg) were dispersed in 7 mL of ethyl alcohol, chilled at 4 °C for 1 h, and subsequently centrifuged at 1200× *g* for 20 min at 4 °C. The resulting supernatant was dried down using nitrogen gas, resuspended in 1 mL of water, and lyophilized for permethylation. Permethylated oligosaccharides were then purified using dichloromethane (DCM) and extracted through centrifugation. The top layer, containing permethylated glycans, was transferred to a clean glass tube, dried under nitrogen, and subjected to structural analysis using MALDI-TOF MS (AB SCIEX TOF/TOF 5800 instrument). Analyzed oligosaccharides included hexose (Hex) and pentose (Pen) oligosaccharides, specifically (Hex)3, (Hex)4, (Hex)5, (Hex)6, (Pen)3, (Pen)4, (Pen)5, and (Pen)6. Data on ileal oligosaccharide content were presented both corrected and uncorrected for the titanium marker, and correction was performed using the following formula:

(4) $$Marker=corrected\;oligosaccharide\;content=O_{diet}\times \left[\frac{C_{diet}}{C_{digesta}}\right]$$

C*_diet_* = TiO_2_ content in the diet

C*_digesta_* = TiO_2_ content in the ileal digesta

O*_diet_* = oligosaccharide content in the diet

### 2.4. Statistical Analysis

Data were analyzed as a 3 × 2 factorial using the PROC GLM procedure of SAS (version 9.4, SAS Institute Inc., Cary, NC, USA). The factors included wheat bran (0%, 5% coarse WB, 5% fine WB) and stimbiotic supplementation (0 or 100g/ton of feed). Significance was set at *p* ≤ 0.05 and tendency was declared at 0.05 < *p* ≤ 0.1. Significantly different means were separated using Tukey’s HSD.

## 3. Results

### 3.1. Growth Performance of Broilers in Response to Dietary Supplementation of Stimbiotics or Wheat Bran Inclusion

There was a significant main effect of WB (*p* < 0.01) and STB supplementation (*p* < 0.005) on feed intake and the FCR during starter phase (Table 6). Feed intake and the FCR were increased with coarse or fine-WB inclusion compared to diets without either of these, whereas the main effect of STB supplementation increased feed intake (*p* < 0.01) and the FCR (*p* = 0.013). There was a tendency for interaction between STB supplementation and WB for weight gain (*p* = 0.053). Supplementation with STBs had no effect on weight gain in broilers receiving diets without WB or in fine-WB-based diets; however, STB supplementation decreased weight gain in coarse-WB-based diets (*p* = 0.120).

There was an interaction between factors for feed intake from day 0 to 28, where STB supplementation had no effect on feed intake in diets without WB, whereas STB supplementation to coarse WB diets tended to decrease feed intake but increased feed intake in broilers fed fine-WB-based diets (*p* = 0.036). There was only a tendency for a significant main effect of WB on weight gain (*p* = 0.084), where coarse WB supplementation tended to decrease weight gain, but fine-WB inclusion tended to increase weight gain. There was a significant main effect of WB on FCR (*p* = 0.047) where coarse WB inclusion increased FCR compared to diets without any WB or with fine WB. 

For the overall phase (day 0 to 42), a tendency was observed for WB (*p* = 0.086) in relation to feed intake, where there was a decrease in feed intake in broilers receiving coarse-WB-based diets compared to diets without WB or fine-WB-based diets. There was a significant interaction between factors for weight gain (*p* = 0.023); STB supplementation tended to decrease weight gain in diets without WB or diets with coarse WB compared to fine WB. There was no significant main effect nor interaction between factors for the FCR. 

### 3.2. Digesta pH in Response to Dietary Supplementation of Stimbiotic or Wheat Bran Inclusion

There were no significant interactions nor main effects between the factors for jejunum and gizzard pH measured at day 18 (Table 7). There was only a tendency for a significant main effect of STB supplementation on cecal pH (*p* = 0.067), where STB supplementation tended to decrease the pH. There was a significant interaction between WB and STB supplementation for jejunum pH determined at day 42 (*p* = 0.042), where in diets without WB, supplementation with STB had only a tendency for a decrease in the jejunum digesta pH. Supplementation with STB of coarse- or fine-WB-based diets decreased the jejunum pH compared to diets without STB supplementation (*p* = 0.001). There was a significant main effect of STB supplementation of a decreased gizzard pH compared to diets without STB supplementation. There was no significant interaction nor main effects between factors for cecal pH. 

### 3.3. Ileal Nutrient Digestibility in Response to Dietary Supplementation of Stimbiotics or Wheat Bran Inclusion

There were no significant interactions nor significant main effects for DMD determined at day 18 (Table 8). There was a significant main effect of WB on ND (*p* = 0.010) and IDE (*p* < 0.01), where fine-WB inclusion increased ND and IDE compared to coarse-WB inclusion or diets without WB. There was no significant interaction nor significant main effect between factors for DMD and ND determined at day 42. There was only a significant main effect of STB supplementation on IDE (*p* = 0.011), where STB supplementation increased the IDE compared to diets without STB supplementation. 

### 3.4. Jejunum Histomorphology in Response to Dietary Supplementation of Stimbiotics or Wheat Bran Inclusion

There was no significant interaction between factors for VH, CD, VW, and VH:CD determined at day 18 and day 42 (Table 9). There was a tendency for a significant main effect of WB inclusion (*p* = 0.051) where fine WB increased the VH compared to diets without WB and diets with coarse-WB inclusion. A significant main effect of STB supplementation (*p* = 0.048) increased VH in broiler chickens receiving STB supplementation compared to those without STB supplementation. There was no significant main effect of WB or STB supplementation for CD, VW, and VH:CD determined at day 18. There was no significant main effect of WB or STB supplementation for VH, CD, VW, and VH:CD determined at day 42, except for a tendency for an increase in VW (*p* = 0.076) in broilers receiving fine-WB-based diets compared to coarse WB or diets without WB. 

### 3.5. Jejunum mRNA Expression of Nutrient Transporters in Response to Dietary Supplementation of Stimbiotics or Wheat Bran Inclusion

There were no significant significant main effects nor interactions between factors for glucose and amino acid transporters determined both on day 18 (Table 10).

There were no significant main effects nor significant interactions between factors for glucose and amino acid transporters determined on day 42 (Table 11). There was only a tendency for an interaction between factors for the mRNA expression of GLUT-5 (*p* = 0.057). Supplementation with STBs or WB inclusion tended to decrease the mRNA expression of GLUT-5; however, STB supplementation to coarse WB tended to increase mRNA expression. 

### 3.6. Profile of Cecal Short-Chain Fatty Acids in Response to Dietary Supplementation of Stimbiotics or Wheat Bran Inclusion

There were no significant interactions nor main effects between factors for the cecal concentrations of acetate, propionate, butyrate, isovalerate, and valerate determined at day 18 (Table 12). There was only a tendency for an interaction between factors for acetate (*p* = 0.061), where fine-WB inclusion tended to decrease the acetate concentration compared to coarse WB or diets without WB in diets not supplemented with STBs. However, in diets supplemented with STBs, fine WB tended to increase the acetate concentration. The significant main effect of WB on isobutyrate (*p* = 0.015) was that it decreased its concentration with coarse or fine-WB inclusion compared to diets without WB. There was only a tendency for a decrease in isobutyrate (*p* = 0.086) and isovalerate (*p* = 0.081) with STB supplementation. There was a significant interaction between factors for the total SCFA (*p* = 0.045). Supplementation with STB had no effect on the total SCFA in diets without WB. However, STB supplementation of coarse WB tended to decrease the total SCFA, while in fine-WB-based diets, it tended to increase the cecal total SCFA concentration (*p* = 0.045). The total BCFA concentration was significantly decreased with dietary WB inclusion, regardless of WB particle size. 

There were no significant main effects nor significant interactions between factors for the cecal concentration of acetate, propionate, isobutyrate, butyrate, isovalerate, and valerate determined on day 42 (Table 13). There was only a tendency for a decrease in the cecal concentrations of isobutyrate (*p* = 0.086) and valerate at day 42 (*p* = 0.099) with STB supplementation. 

### 3.7. The Oligosaccharide Profile in the Ileal Digesta of Broiler Chickens Receiving Dietary Supplementation of Stimbiotics or Wheat Bran Inclusion

There was no significant interaction between factors for marker-corrected ileal digesta hexose and pentose oligosaccharides determined on day 42 (Table 14). There was no significant main effect of WB nor significant main effect of STB supplementation on (Hex)3, (Hex)4, (Hex)5, (Pent)4, or (Pent)5. There was a significant main effect of WB (*p* = 0.007) on (Hex)6, where fine-WB inclusion increased its concentration in the ileum compared to coarse WB or diets without WB. There was only a tendency for a significant main effect of STB supplementation (*p* = 0.097) on the (Pent)3 concentration in the ileum. Supplementation with STBs (*p* = 0.097) tended to increase the (Pent)3 concentration in the ileum compared to diets without STB supplementation. 

## 4. Discussion

Moderate inclusion of fibers into broiler diets has been shown to be beneficial; however, there is still no consensus on the effect of the particle size of the fiber. So, the objective of this study was to determine the impact of the particle size of WB and its interactive effect with STB supplementation in broilers. All the experimental diets were formulated to provide similar amounts of nutrients and energy to broilers, but there were differences in the performance of broilers. Coarse or fine WB at a 50 g/kg inclusion level increased the feed intake and FCR of broilers in the starter phase. From the current literature, a decrease or no effect on feed intake was observed in most studies when WB was included at ≤50 g/kg levels into broiler diets [11,21,22], whereas inclusion of WB at >50 g/kg or more into broiler diets increased feed intake and the FCR [23,24,25,26,27]. Our data suggest that the current inclusion rate of WB was at the threshold and mimicked the responses where more than 50 g/kg WB was used. Increased fiber inclusion levels at more than 50 g/kg significantly increase the fiber level in the diet, which can reduce feed intake by increasing the digesta viscosity and bulkiness, and decreasing the digesta flow rate. However, at an inclusion level of less than 50 g/kg, the level of fiber may not be sufficient to decrease the digesta flow rate, so an increase in feed intake was not usually observed at this inclusion level. 

At a 50 g/kg inclusion level of WB, the effect on broiler growth performance is not apparent. In the current study, at a 50 g/kg inclusion level, the antinutritive effects of either coarse or fine WB were observed for the FCR of broiler chickens during the starter phase. However, during the grower phase, the negative effect of feeding coarse, but not fine, WB was manifested as decreased nutrient digestibility and villi height. So, this suggests that during the starter phase, broilers try to maintain their weight gain by increasing their feed intake, which increases the FCR both with the coarse or fine particle size of the fiber. However, during the grower phase, the particle size of the fiber makes a huge difference in how broilers react to their inclusion. Grower-phase broiler chickens fed fine-WB-based diets were able to maintain their weight gains without increasing their feed intake. Reducing the particle size of the WB results in a low water-holding capacity compared to the coarse particle size, which also reduces the viscosity of the digesta [9]. One possible reason for no negative effect on feed intake and weight gain in grower-phase broiler chockens due to fine-wheat-bran inclusion could be attributed to (1) an adaptation to fiber, (2) an increase in nutrient digestibility, and (3) an increase in villi height. An increase in villi height increases the surface area for the absorption of nutrients, which likely partly explains the increase in ileal digestibility of energy and nitrogen observed in this study, which led to a decrease in the FCR. Along a similar line, an increase in starter-phase FCR and feed intake, was observed with STB supplementation only in starter-phase broilers but not in grower-phase broilers, and similar effects have been reported in previous studies [28,29]. A previous study from our lab [22] also found no effects on the performance of broilers with STB supplementation (with or without WB inclusion) in starter-phase (day 0 to 10) broilers. There is no clear explanation for such an effect but it could be due to the adaptation or immaturity of the gut of young broilers to utilize STB fully and beneficially.

An increase in the nutrient digestibility and jejunum villi height on day 18 with fine-WB inclusion did not translate into an increase in weight gain in the corresponding grower phase. A tendency for a numerical increase in body-weight gains were observed with fine-WB inclusion compared to coarse-WB inclusion. This could be because the coarse particle size of any fiber was shown to increase the relative weights of gizzards, which may translate to an increase in weight gain [30,31]. But, the relative weight of the gizzard decreases with a decrease in the particle size of the fiber [32]. In contrast, fine WB increased the weight gains by increasing nutrient digestibility. In a study by Novotný et al. [33] an increase in gizzard weight with coarse-particle-size feed was observed, while a decrease was observed with fine particle size of the feed. In the same study, the overall body-weight gain of broilers receiving coarse- or fine-particle-size WB was not different, but the carcass weight was increased by 9% in fine- compared to coarse-particle-size feed. It is possible that the increased gizzard weight of birds receiving diets containing coarse wheat bran is the reason why the birds in those groups were heavier. To clearly differentiate the mechanism of the increase in body-weight gain with the coarse or fine particle size of fiber inclusion, one should determine the weight gains without the gizzard, or weights after processing. 

An increase in hexose and pentose oligosaccharides were observed with WB inclusion and a tendency for a further increase was observed with fine-WB inclusion. Although not significant, there was a numerical increase in (Pent)3 concentration in the ileal digesta with WB when supplemented with STB. As hypothesized, reducing the particle size of WB increases the surface area for the enzyme component of the STB to act on and increases the concentration of ileal oligosaccharides. Supplementation with STB did not have any effect on the total SCFA in diets without WB. There was no clear understanding of why a decrease in the total SCFA was observed with fine-WB inclusion. However, a decrease in total SCFA with fine-WB inclusion was reversed with STB supplementation. The fine particle size of the WB increases the surface for the STB to act on and facilitates the hydrolysis of complex fiber into oligosaccharides [11]. 

The current hypothesis is that oligosaccharides released in the ileal digesta with fine WB will be utilized in the ceca and this leads to an increase in concentration of SCFA. Contrary to expectations, no such effect was observed on the concentration of SCFA in the ceca. This could be due to two reasons: (1) these oligosaccharides might have been already fermented by microbes in the ileum or (2) they could have been fermented in the ceca, leading to the release of short-chain fatty acids. However due to their rapid absorption [34,35], an increase in concentration was not observed. However, during day 18 or 42, there was a decrease in the concentration of isobutyrate and isovalerate (which belong to the branched-chain fatty acids) either with WB inclusion or with STB supplementation. A similar trend was observed in a similar study reported earlier [22]. A reduction in BCFA levels indicates decreased protein fermentation, suggesting that less protein was being fermented in the ceca, either due to enhanced protein utilization in the upper gastrointestinal tract (GIT) resulting in less protein to ferment, or increased levels or fermentable fiber, which is preferentially fermented over protein. The beneficial effects of a decrease in BCFAs include a reduction in the production of harmful byproducts, which have negative effect of gut health of broilers [36,37,38]. A decrease in the pH of the gizzard, jejunum, and cecum with STB supplementation potentially reduces the growth of pathogenic bacteria and increases the secretion of digestive enzymes, which increases nutrient digestibility [39,40]. This also partly explains an increase in IDE on day 42 with STB supplementation.

## 5. Conclusions

In conclusion, wheat bran inclusion or stimbiotic supplementation increased the FCR in young broilers, but during the grower or finisher phases, reducing the particle size of the wheat bran along with stimbiotic supplementation had no negative effect in terms of performance and nutrient digestibility. Stimbiotic supplementation or coarse- or fine-wheat-bran inclusion did not influence the total short-chain fatty acid concentration.

## Figures and Tables

**Table 1:** Feedstuff and chemical composition (g/kg) of the starter-phase (d 0 to 10) diets.

Items	Control	Control + Coarse WB	Control + Fine WB
Wheat bran (WB)—coarse		50	
Wheat bran—fine			50
Corn	617	560	560
Soybean meal (48%)	325	319	319
Soya oil	21.4	35	35
NaCl	3.6	3.6	3.6
Limestone	4.3	4.6	4.6
Dicalcium phosphate	12.3	11.8	11.8
Lysine HCl	2.3	2.4	2.4
DL-Methionine	2.9	2.9	2.9
Threonine	0.6	0.7	0.7
Valine	0.6	0.7	0.7
Mineral premix ^1^	5.0	5.0	5.0
Vitamin premix ^2^	5.0	5.0	5.0
Quantum Blue 5G ^3^	0.1	0.1	0.1
Total	1000	1000	1000
Calculated nutrient content, g/kg			
Crude protein	215	215	215
ME kcal/kg	3050	3050	3050
Dry matter	870	872	872
Ca	9.0	9.0	9.0
Total P	7.7	8.0	7.0
Non-phytate P	5.1	5.1	5.1
Digestible amino acids, g/kg			
Methionine	6.0	6.1	6.1
Cysteine	3.7	3.7	3.7
Lysine	13.4	13.4	13.4
Threonine	8.7	8.7	8.7
Tryptophan	2.5	2.6	2.6
Arginine	14.1	14.2	14.2
Valine	10.4	10.4	10.4
Isoleucine	8.8	8.8	8.8
Analyzed nutrient content			
DM	880	880	880
Crude protein	220	220	220
Phytate P	3.0	3.1	3.1
Acid detergent fiber	34.2	36.3	36.3
Neutral detergent fiber	73.8	89.6	89.6

^1^ Vitamin A, 5484 IU; vitamin D3, 2643 ICU; vitamin E, 11 IU; menadione sodium bisulfate, 4.38 mg; riboflavin, 5.49 mg; d-pantothenic acid, 11 mg; niacin, 44.1 mg; choline chloride, 771 mg; vitamin B12, 13.2 µg; biotin, 55.2 µg; thiamine mononitrate, 2.2 mg; folic acid, 990 µg; pyridoxine hydrochloride, 3.3 mg. ^2^ Iodine, 1.11 mg; manganese, 66.06 mg; copper, 4.44 mg; iron, 44.1 mg; zinc, 44.1 mg; selenium, 300 µg. ^3^ Quantum Blue phytase supplemental dose of 500 FTU/kg of feed.

**Table 2:** Feedstuff and chemical composition (g/kg) of the grower-phase (d 10 to 28) diets.

Items	Control	Control + Coarse WB	Control + Fine WB
Wheat bran (WB)—coarse		50	
Wheat bran—fine			50
Corn	683	626	626
Soybean meal (48%)	261	255	255
Soya oil	20	33	33
NaCl	3.7	3.7	3.7
Limestone	4.1	4.4	4.4
Dicalcium Phosphate	10.9	10.4	10.4
Lysine HCl	2.9	2.9	2.9
DL-Methionine	2.5	2.6	2.6
Threonine	0.7	0.7	0.7
Valine	0.8	0.8	0.8
Mineral premix ^1^	5.0	5.0	5.0
Vitamin premix ^2^	5.0	5.0	5.0
Quantum Blue 5G ^3^	0.1	0.1	0.1
Total	1000	1000	1000
Calculated nutrient content, g/kg			
Crude protein	190	190	190
ME, kcal/kg	3100	3100	3100
Dry matter	869	870	870
Ca	8.4	8.4	8.4
Total P	7.1	7.4	7.4
Non-phytate P	4.7	4.6	4.6
Digestible amino acids, g/kg			
Methionine	5.4	5.4	5.4
Cysteine	3.4	3.4	3.4
Lysine	12.0	12.1	12.1
Threonine	7.8	7.8	7.8
Tryptophan	2.2	2.2	2.2
Arginine	12.0	12.1	12.1
Valine	9.4	9.4	9.4
Isoleucine	7.6	7.6	7.6
Analyzed nutrient content, g/kg			
Dry matter	890	890	890
Crude protein	170	160	160
Phytate P	2.6	2.7	2.7
Acid detergent fiber	37.0	36.5	36.5
Neutral detergent fiber	78.2	85.9	85.9

^1^ Vitamin A, 5484 IU; vitamin D3, 2643 ICU; vitamin E, 11 IU; menadione sodium bisulfate, 4.38 mg; riboflavin, 5.49 mg; d-pantothenic acid, 11 mg; niacin, 44.1 mg; choline chloride, 771 mg; vitamin B12, 13.2 µg; biotin, 55.2 µg; thiamine mononitrate, 2.2 mg; folic acid, 990 µg; pyridoxine hydrochloride, 3.3 mg. ^2^ Iodine, 1.11 mg; manganese, 66.06 mg; copper, 4.44 mg; iron, 44.1 mg; zinc, 44.1 mg; selenium, 300 µg. ^3^ Quantum Blue phytase equivalent to the supplemental dose of 500 FTU/kg of feed.

**Table 3:** Feedstuff and chemical composition (g/kg) of finisher-phase (d 28 to 42) diets.

Items	Control	Control + Coarse WB	Control + Fine WB
Wheat bran (WB)—coarse		50	
Wheat bran—fine			50
Corn	704	647	647
Soybean meal (48%)	241	234	234
Soya oil	25	38	38
NaCl	3.7	3.7	3.7
Limestone	3.7	4.0	4.0
Dicalcium phosphate	8.5	8.0	8.0
Lysine HCl	2.0	2.1	2.1
DL-Methionine	2.1	2.2	2.2
Threonine	0.5	0.5	0.5
Valine	0.1	0.2	0.2
Mineral premix ^1^	5.0	5.0	5.0
Vitamin premix ^2^	5.0	5.0	5.0
Quantum Blue 5G ^3^	0.1	0.1	0.1
Total	1000	1000	1000
Calculated nutrient content, g/kg			
Crude protein	180	180	180
ME, kcal/kg	3150	3150	3150
Dry matter	869	871	871
Calcium	7.6	7.6	7.6
Total P	6.6	6.9	6.9
Non-phytate P	4.2	4.1	4.1
Phytate	2.4	2.8	2.8
Digestible amino acids, g/kg			
Methionine	4.9	4.9	4.9
Cysteine	3.3	3.3	3.3
Lysine	10.8	10.8	10.8
Threonine	7.3	7.3	7.3
Tryptophan	2.0	2.1	2.1
Arginine	11.4	11.5	11.5
Valine	8.3	8.4	8.4
Isoleucine	7.2	7.2	7.2
Analyzed nutrient content, g/kg			
Dry matter	880	880	880
Crude protein	160	160	160
Phytate P	2.7	2.8	2.8
Acid detergent fiber	36.0	37.5	37.5
Neutral detergent fiber	75.9	83.8	83.8

^1^ Vitamin A, 5484 IU; vitamin D3, 2643 ICU; vitamin E, 11 IU; menadione sodium bisulfate, 4.38 mg; riboflavin, 5.49 mg; d-pantothenic acid, 11 mg; niacin, 44.1 mg; choline chloride, 771 mg; vitamin B12, 13.2 µg; biotin, 55.2 µg; thiamine mononitrate, 2.2 mg; folic acid, 990 µg; pyridoxine hydrochloride, 3.3 mg. ^2^ Iodine, 1.11 mg; manganese, 66.06 mg; copper, 4.44 mg; iron, 44.1 mg; zinc, 44.1 mg; selenium, 300 µg. ^3^ Quantum Blue phytase equivalent to the supplemental dose of 500 FTU/kg of feed.

**Table 4:** Analyzed enzyme activity of the diets.

Diet Phase	Diet	Xylanase (BXU ^1^/kg)	Phytase (FTU ^2^/kg)
Starter	Basal	<2000	964
	Basal + Coarse WB	<2000	875
	Basal + Fine WB	<2000	1050
	Basal + STB	19,800	870
	Basal + Coarse WB+ STB	22,200	1100
	Basal + Fine WB+ STB	22,700	1040
Grower	Basal	<2000	950
	Basal + Coarse WB	<2000	888
	Basal + Fine WB	<2000	1150
	Basal + STB	15,600	843
	Basal + Coarse WB+ STB	16,000	928
	Basal + Fine WB+ STB	16,300	1040
Finisher	Basal	<2000	849
	Basal + Coarse WB	<2000	1290
	Basal + Fine WB	<2000	1220
	Basal + STB	12,500	960
	Basal + Coarse WB+ STB	14,300	991
	Basal + Fine WB+ STB	16,400	1010

^1^ One unit of BXU is defined as the amount of enzyme that produces 1 nmol of reducing sugar from xylan as xylo-oligosaccharidese at pH 5.3 and 50 °C. ^2^ One unit of FTU is defined as the amount of enzyme required to release 1 µmol of inorganic phosphorus per minute from sodium phytate at pH 5.5 and 37 °C.

**Table 5:** List of primers used for qRT-PCR.

Gene	Full Name	Function	Forward Primer	Reverse Primer
-actin	Beta-actin	Housekeeping gene	CAACACAGTGCTGTCTGGTGGTA	ATCGTACTCCTGCTTGCTGATCC
b^0+^AT	Solute carrier family 7-member 9	Na+-independent neutral/cysteine, cationic amino acid exchanger	CAGTAGTGAATTCTCTGAGTGTGAAGCT	GCAATGATTGCCACAACTACCA
GLUT-1	Glucose Transporter 1	Glucose transporter	CTTTGTCAACCGCTTTGG	CAGAATACAGGCCG ATGAT
GLUT-2	Glucose transporter 2	Glucose transporter	TTCATTGTAGCTGAGCTGTT	CGAAGACAACGAACACATAC
GLUT-5	Glucose transporter 5	Glucose transporter	TTGCTGGCTTTGGGTTGTG	GGAGGTTGAGGGCCAAAGTC
rBAT	Solute carrier family 3 member 1	Dimerize with b^o,+^AT	CCCGCCGTTCAACAAGAG	AATTAAATCCATCGACTCCTTTGC
pepT-1	Peptide transporter 1	Peptide transporter	CCCCTGAGGAGGATCACTGTT	CAAAAGAGCAGCAGCAACGA
SGLT-1	Sodium glucose transporter 1	Glucose transporter	GCCGTGGCCAGGGCTTA	CAATAACCTGATCTGTGACCAGT
SGLT-4	Sodium glucose transporter 4	Glucose transporter	ATACCCAAGGTAATAGTCCCAAAC	TGGGTCCCTGAACAAATGAAA
y+ LAT1	y+ L amino acid transporter 1	Na+-dependent neutral/cationic amino acid exchanger	CAGAAAACCTCAGAGCTCCCTTT	TGAGTACAGAGCCAGCGCAAT
CAT2	Cationic amino acid transporter 2	Amino acid transporter	TGGATCAGGTTTAGCATCTG	CGGAACAAGAATCTCCATCT

**Table 6:** Influence of wheat bran particle size and stimbiotic supplementation on growth performance of broiler chickens.

WB, g/kg	STB	Day 0–10			Day 0–28			Day 0–42		
		FI, g	Gain, g	FCR	FI, g	Gain, g	FCR	FI, g	Gain, g	FCR
Means for main effect of wheat bran										
0		249 ^b^	186	1.34 ^b^	2167	1514	1.43 ^b^	5074	2935	1.74
50 g/kg Coarse		278 ^a^	187	1.50 ^a^	2149	1471	1.46 ^a^	4901	2887	1.70
50 g/kg Fine		289 ^a^	195	1.48 ^a^	2229	1558	1.43 ^b^	5103	2922	1.76
Pooled SEM		4.67	3.46	0.04	29	26	0.01	65	62	0.04
*p*-Value		<0.001	0.120	0.013	0.144	0.084	0.047	0.086	0.789	0.486
Means for main effect of STB										
	−	264	190	1.40	2191	1516	1.45	5041	2944	1.72
	+	280	189	1.49	2173	1513	1.44	5010	2886	1.74
Pooled SEM		3.81	2.82	0.03	23	21	0.01	53	51	0.03
*p*-Value	STB	0.005	0.792	0.046	0.606	0.918	0.491	0.689	0.328	0.590
Means for interaction effects										
0	−	244	184 ^a^	1.33	2155 ^ab^	1485	1.45	5078	3014 ^a^	1.69
50 g/kg Coarse	−	272	193 ^a^	1.41	2221 ^ab^	1521	1.46	5018	2986 ^ab^	1.69
50 g/kg Fine	−	276	191 ^a^	1.45	2195 ^ab^	1541	1.43	5028	2832 ^ab^	1.79
0	+	255	188 ^a^	1.36	2180 ^ab^	1544	1.41	5070	2856 ^ab^	1.79
50 g/kg Coarse	+	284	179 ^b^	1.60	2077 ^b^	1420	1.46	4783	2789 ^b^	1.72
50 g/kg Fine	+	302	199 ^a^	1.52	2262 ^a^	1574	1.44	5177	3012 ^a^	1.73
Pooled SEM		6.60	4.89	0.06	40	37	0.01	92	88	0.05
*p*-Value		0.423	0.053	0.378	0.036	0.089	0.146	0.142	0.023	0.257

WB, wheat bran; STB, stimbiotic; FI, feed intake; FCR, feed conversion ratio. *n* = 16, 24, and 6 for the main effect means of WB, STB, and interaction effect means, respectively. ^a,b^ Means in a column, within a group, with different superscripts are significantly different (*p* < 0.05).

**Table 7:** Influence of wheat bran particle size and stimbiotic supplementation on pH of gastrointestinal tract in broiler chickens.

WB, g/kg	STB	Day 18			Day 42		
		Jejunum	Gizzard	Cecum	Jejunum	Gizzard	Cecum
Means for main effect of wheat bran							
0		6.34	3.48	6.30	6.56	3.53	7.52
50 g/kg Coarse		6.33	3.40	6.26	6.50	3.50	7.44
50 g/kg Fine		6.41	3.26	6.29	6.53	3.46	7.47
Pooled SEM		0.038	0.146	0.105	0.037	0.077	0.080
*p*-Value		0.294	0.585	0.883	0.579	0.817	0.742
Means for main effect of STB							
	−	6.37	3.39	6.38	6.61	3.64	7.55
	+	6.35	3.37	6.18	6.45	3.35	7.40
Pooled SEM		0.031	0.120	0.086	0.031	0.063	0.066
*p*-Value		0.666	0.938	0.067	0.001	0.002	0.108
Means for interaction effects							
0	−	6.34	3.57	6.40	6.56 ^a^	3.73	7.62
50 g/kg Coarse	−	6.33	3.41	6.35	6.62 ^a^	3.67	7.44
50 g/kg Fine	−	6.44	3.18	6.38	6.66 ^a^	3.53	7.60
0	+	6.33	3.39	6.19	6.56 ^ab^	3.33	7.43
50 g/kg Coarse	+	6.34	3.39	6.15	6.39 ^c^	3.33	7.43
50 g/kg Fine	+	6.38	3.34	6.21	6.41 ^bc^	3.40	7.34
Pooled SEM		0.05	0.21	0.15	0.05	0.11	0.11
*p*-Value		0.779	0.719	0.987	0.042	0.441	0.540

WB, wheat bran; STB, stimbiotic. *n* = 16, 24, and 6 for the main effect means of WB, STB, and interaction effect means, respectively. ^a–c^ Means in a column, within a group, with different superscripts are significantly different (*p* < 0.05).

**Table 8:** Influence of wheat bran particle size and stimbiotic supplementation on apparent ileal nutrient digestibility in broiler chickens.

WB, g/kg	STB	Day 18			Day 42		
		DMD%	ND%	IDE, MJ/kg	DMD%	ND%	IDE, MJ/kg
Means for main effect of wheat bran							
0		70.5	76.2 ^b^	13.36 ^b^	73.3	80.2	12.21
50 g/kg Coarse		70.0	77.3 ^b^	13.59 ^b^	73.6	81.2	12.10
50 g/kg Fine		71.7	79.2 ^a^	14.39 ^a^	74.6	82.5	12.48
Pooled SEM		0.89	0.66	0.159	0.84	0.86	0.120
*p*-Value		0.441	0.010	< 0.01	0.614	0.188	0.177
Means for main effect of STB							
	−	70.1	77.0	13.74	73.4	80.9	12.04
	+	71.4	78.1	13.82	74.2	81.7	12.47
Pooled SEM		0.73	0.54	0.130	0.68	0.70	0.098
*p*-Value	STB	0.176	0.133	0.684	0.399	0.491	0.011
Means for interaction effects							
0	−	68.7	74.4	13.09	72.5	79.6	11.96
50 g/kg Coarse	−	70.0	77.6	13.68	73.3	80.9	11.95
50 g/kg Fine	−	71.6	79.0	14.46	74.5	82.1	12.25
0	+	72.4	78.0	13.64	74.1	80.7	12.43
Coarse	+	70.0	77.0	13.50	73.9	81.5	12.28
Fine	+	71.9	79.4	14.32	74.7	82.8	12.64
Pooled SEM		1.26	0.93	0.225	1.19	1.21	0.169
*p*-Value		0.252	0.088	0.202	0.762	0.973	0.923

STB, stimbiotic; DMD, dry mater digestibility; ND, nitrogen digestibility; IDE, ileal digestible energy. *n* = 16, 24, and 6 for the main effect means of WB, STB, and interaction effect means, respectively. ^a,b^ Means in a column, within a group, with different superscripts are significantly different (*p* < 0.05).

**Table 9:** Influence of wheat bran particle size and stimbiotic supplementation on jejunum histomorphology in broiler chickens.

WB, g/kg	STB	Day 18				Day 42			
		VH, *μ*m	CD, *μ*m	VW, *μ*m	VH/CD	VH, *μ*m	CD, *μ*m	VW, *μ*m	VH/CD
Means for main effect of wheat bran									
0		1230 ^b^	145	157	8.70	1596	180	140	10.95
50 g/kg Coarse		1248 ^b^	153	154	8.53	1590	146	138	11.09
50 g/kg Fine		1346 ^a^	147	156	9.18	1629	155	153	10.94
Pooled SEM		34.3	7.23	5.07	0.37	59.3	20.9	4.64	0.66
*p*-Value		0.051	0.546	0.912	0.457	0.881	0.499	0.076	0.984
Means for main effect of STB									
	−	1235	146	156	8.67	1603	175	145	10.69
	+	1314	151	155	8.93	1607	146	142	11.29
Pooled SEM		28.0	5.91	4.14	0.31	48.5	17.1	3.79	0.54
*p*-Value		0.048	0.749	0.899	0.553	0.955	0.236	0.647	0.430
Means for interaction effects									
0	−	1212	141	155	8.85	1557	225	145	9.53
50 g/kg Coarse	−	1233	154	162	8.35	1563	142	139	11.11
50 g/kg Fine	−	1260	143	150	8.82	1689	156	151	11.43
0	+	1249	149	158	8.56	1634	134	136	12.36
50 g/kg Coarse	+	1263	152	145	8.70	1617	149	137	11.07
50 g/kg Fine	+	1431	152	162	9.53	1570	154	154	10.45
Pooled SEM		55.0	11.8	8.28	0.61	96.9	34.2	7.57	1.08
*p*-Value		0.292	0.825	0.128	0.640	0.451	0.200	0.687	0.116

STB, stimbiotic; VH, villi heigh; CD, crypt depth; V, villi width. *n* = 16, 24, and 6 for the main effect means of WB, STB, and interaction effect means, respectively.

**Table 10:** Influence of wheat bran particle size and stimbiotic supplementation on jejunum expression of nutrient transporters in broiler chickens at day 18.

WB, g/kg	STB	b^0+^AT	GLUT-1	GLUT-2	GLUT-5	rBAT	pepT-1	SGLT-1	SGLT-4	y+ LAT1	CAT2
Means for main effect of wheat bran											
0		1.18	1.21	1.33	2.34	0.55	1.01	1.95	2.42	1.79	1.22
50 g/kg Coarse		0.99	0.84	0.84	0.88	0.01	0.94	0.93	0.71	0.90	1.02
50 g/kg Fine		0.96	0.73	0.94	1.07	0.03	0.99	0.92	0.89	0.90	1.84
Pooled SEM		0.133	0.209	0.194	0.678	0.244	0.104	0.489	0.879	0.483	0.579
*p*-Value		0.522	0.295	0.244	0.401	0.398	0.891	0.312	0.403	0.395	0.630
Means for main effect of STB											
	−	0.93	0.85	0.83	0.98	0.32	0.91	0.94	0.84	0.94	0.99
	+	1.15	1.02	1.25	1.80	0.01	1.05	1.61	1.81	1.44	1.73
Pooled SEM		0.109	0.171	0.159	0.553	0.199	0.085	0.399	0.718	0.394	0.473
*p*-Value	STB	0.224	0.598	0.102	0.343	0.298	0.332	0.311	0.387	0.407	0.315
Means for interaction effects											
0	−	1.00	1.00	1.00	1.00	1.00	1.00	1.00	1.00	1.00	1.00
50 g/kg Coarse	−	1.00	0.88	0.71	0.81	0.01	0.75	0.84	0.47	0.82	0.82
50 g/kg Fine	−	0.80	0.69	0.79	1.14	0.06	1.00	0.98	1.11	1.00	1.13
0	+	1.36	1.40	1.62	3.34	0.02	1.02	2.78	3.65	2.48	1.41
50 g/kg Coarse	+	0.97	0.79	0.96	0.96	0.01	1.12	1.02	0.99	0.99	1.21
50 g/kg Fine	+	1.10	0.78	1.11	0.99	0.01	0.99	0.85	0.69	0.79	2.55
Pooled SEM		0.188	0.296	0.275	0.958	0.345	0.147	0.692	1.243	0.682	0.818
*p*-Value		0.627	0.739	0.795	0.414	0.415	0.428	0.373	0.479	0.455	0.801

b0+AT, solute carrier family 7-member 9; GLUT-1, glucose tranporter-1; GLUT-2, glucose transporter-2; GLUT-5, glucose transporter-5; rBAT, solute carrier family member 3; pepT-1 – peptide transporter-1; y+ LAT1: Na+-dependent cationic amino acid transporter; SGLT-1: Na–glucose transporter-1; SGLT-4: Na–glucose transporter-4; CAT2: cationic amino acid transporter 2. *n* = 16, 24, and 6 for the main effect means of WB, STB, and interaction effect means, respectively.

**Table 11:** Influence of wheat bran particle size and stimbiotic supplementation on jejunum expression of nutrient transporters in broiler chickens at day 42.

WB, g/kg	STB	b0+AT	GLUT-1	GLUT-2	GLUT-5	rBAT	pepT-1	SGLT-1	SGLT-4	y+LAT1	CAT2
Means for main effect of wheat bran											
0		0.99	1.04	0.98	0.86	1.02	1.02	1.05	1.04	1.09	0.92
50 g/kg Coarse		0.89	1.00	0.86	0.83	1.09	1.42	1.12	1.06	1.12	0.82
50 g/kg Fine		0.95	0.94	0.94	0.74	0.97	1.38	1.01	1.17	1.12	0.83
Pooled SEM		0.093	0.103	0.069	0.066	0.069	0.133	0.078	0.094	0.079	0.114
*p*-Value		0.779	0.798	0.496	0.571	0.592	0.125	0.742	0.649	0.990	0.755
Means for main effect of STB											
	−	0.99	0.97	0.93	0.85	1.01	1.20	0.99	1.07	1.09	0.83
	+	0.90	1.01	0.92	0.77	1.03	1.34	1.12	1.11	1.12	0.89
Pooled SEM		0.076	0.084	0.056	0.054	0.057	0.108	0.063	0.076	0.065	0.093
*p*-Value	STB	0.454	0.746	0.964	0.418	0.808	0.421	0.151	0.785	0.838	0.677
Means for interaction effects											
0	−	1.00	1.00	1.00	1.00	1.00	1.00	1.00	1.00	1.00	1.00
50 g/kg Coarse	−	0.89	0.99	0.81	0.71	1.02	1.27	0.93	1.07	1.08	0.59
50 g/kg Fine	−	1.07	0.92	0.97	0.79	1.02	1.33	1.03	1.16	1.21	0.87
0	+	0.98	1.08	0.96	0.69	1.04	1.04	1.11	1.09	1.20	0.84
50 g/kg Coarse	+	0.89	1.00	0.90	0.91	1.14	1.54	1.28	1.05	1.14	1.00
50 g/kg Fine	+	0.84	0.96	0.91	0.69	0.92	1.41	1.00	1.18	1.02	0.80
Pooled SEM		0.132	0.145	0.098	0.094	0.098	0.188	0.110	0.132	0.112	0.161
*p*-Value		0.675	0.972	0.751	0.057	0.613	0.837	0.282	0.934	0.256	0.222

b0+AT, solute carrier family 7-member 9; GLUT-1, glucose tranporter-1; GLUT-2, glucose transporter-2; GLUT-5, glucose transporter-5; rBAT, solute carrier family member 3; pepT-1:y+ LAT1, Na+-dependent cationic amino acid transporter/peptide transporter-1; SGLT-1, Na–glucose transporter-1; SGLT-4, Na–glucose transporter-4; CAT2, cationic amino acid transporter 2. *n* = 16, 24, and 6 for the main effect means of WB, STB, and interaction effect means, respectively.

**Table 12:** Influence of wheat bran particle size and stimbiotic supplementation on cecal short-chain fatty acids concentration (mM) in broiler chickens on day 18.

WB, g/kg	STB	Acetate	Propionate	Isobutyrate	Butyrate	Isovalerate	Valerate	Total SCFAs	Total BCFAs
Means for main effect of wheat bran									
0		60.5	4.98	1.00 ^a^	11.57	0.85	1.03	80.6	1.89 ^a^
50 g/kg Coarse		58.1	4.71	0.79 ^b^	11.63	0.73	1.06	78.6	1.52 ^b^
50 g/kg Fine		57.5	4.88	0.84 ^b^	12.03	0.78	0.90	76.2	1.67 ^b^
Pooled SEM		3.245	0.348	0.049	0.987	0.044	0.055	4.4	0.093
*p*-Value		0.813	0.874	0.015	0.970	0.208	0.162	0.789	0.046
Means for main effect of STB									
	−	58.8	5.21	0.93	10.96	0.84	1.05	79.0	1.76
	+	58.8	4.52	0.83	12.43	0.73	0.94	78.1	1.62
Pooled SEM		2.650	0.284	0.040	0.806	0.036	0.045	3.572	0.076
*p*-Value		0.942	0.085	0.086	0.220	0.081	0.130	0.908	0.238
Means for interaction effects									
0	−	60.4	5.49	0.98	10.70	0.82	1.10	80.6 ^ab^	1.78
50 g/kg Coarse	−	64.2	5.41	0.89	12.59	0.83	1.19	88.1 ^a^	1.72
50 g/kg Fine	−	51.5	4.77	0.91	9.58	0.87	0.85	68.2 ^b^	1.78
0	+	60.7	4.47	1.01	12.33	0.88	0.95	80.5 ^ab^	1.97
50 g/kg Coarse	+	52.7	4.02	0.69	10.78	0.63	0.92	70.4 ^ab^	1.32
50 g/kg Fine	+	63.6	5.00	0.78	14.17	0.71	0.96	84.2 ^ab^	1.57
Pooled SEM		4.590	0.491	0.069	1.396	0.062	0.078	6.187	0.132
*p*-Value		0.062	0.251	0.256	0.099	0.112	0.065	0.045	0.096

STB, stimbiotic; SCFA, short-chain fatty acids; BCFA, branched-chain fatty acids. *n* = 16, 24, and 6 for the main effect means of WB, STB, and interaction effect means, respectively. ^a,b^ Means in a column, within a group, with different superscripts are significantly different (*p* < 0.05).

**Table 13:** Influence of wheat bran particle size and stimbiotic supplementation on cecal short-chain fatty acids concentration (mM) in broiler chickens on day 42.

WB, g/kg	STB	Acetate	Propionate	Isobutyrate	Butyrate	Isovalerate	Valerate	Total SCFAs	Total BCFAs
Means for main effect of wheat bran									
0		89.5	4.58	0.43	21.5	0.33	1.05	114	0.76
50 g/kg Coarse		84.1	4.15	0.42	19.7	0.33	1.04	110	0.71
50 g/kg Fine		84.5	4.08	0.38	19.5	0.32	0.86	110	0.67
Pooled SEM		4.2	0.383	0.058	1.371	0.047	0.075	4.939	0.107
*p*-Value		0.611	0.624	0.763	0.482	0.958	0.192	0.837	0.807
Means for main effect of STB									
	−	86.14	4.52	0.48	21.24	0.36	1.05	114	0.80
	+	86.05	4.02	0.35	19.21	0.29	0.91	109	0.63
Pooled SEM		3.463	0.313	0.047	1.120	0.038	0.061	4.033	0.087
*p*-Value		0.963	0.272	0.086	0.189	0.235	0.099	0.395	0.193
Means for interaction effects									
0	−	92.82	4.77	0.53	23.38	0.37	1.17	123	0.90
50 g/kg Coarse	−	79.35	4.75	0.48	19.33	0.39	1.13	105	0.77
50 g/kg Fine	−	86.26	4.04	0.42	21.02	0.33	0.88	112	0.75
0	+	86.19	4.38	0.36	19.68	0.29	0.91	104	0.65
50 g/kg Coarse	+	88.75	3.55	0.37	20.03	0.28	0.95	114	0.65
50 g/kg Fine	+	82.80	4.13	0.33	17.50	0.31	0.84	108	0.60
Pooled SEM		5.999	0.542	0.082	1.939	0.066	0.106	6.985	0.151
*p*-Value		0.380	0.497	0.906	0.455	0.800	0.645	0.153	0.915

STB, stimbiotic; SCFAs, short-chain fatty acids; BCFAs, branched-chain fatty acids. *n* = 16, 24, and 6 for the main effect means of WB, STB, and interaction effect means, respectively.

**Table 14:** Influence of wheat bran particle size and stimbiotic supplementation on marker-corrected ileal digesta hexose and pentose oligosaccharides (*μ*g/100g DM intake) on day 42 of age of the broiler chickens.

WB, g/kg	STB	(Hex)3	(Hex)4	(Hex)5	(Hex)6	(Pent)3	(Pent)4	(Pent)5
Means for main effects of wheat bran								
0		409	1233	218	25 ^b^	58	20	28
50 g/kg Coarse		662	1303	251	74 ^ab^	66	34	45
50 g/kg Fine		483	1240	244	112 ^a^	119	23	30
Pooled SEM		106	268	48.0	16.2	18.8	6.12	7.57
*p*-Value		0.254	0.969	0.874	0.007	0.109	0.317	0.272
Means for main effect of STB								
	−	544	1210	248	70	56	20	40
	+	493	1294	228	77	101	30	28
Pooled SEM		86.2	219	39.2	13.3	15.4	5.00	6.18
*p*-Value		0.715	0.832	0.713	0.978	0.097	0.241	0.209
Means for interaction effects								
0	−	266	1296	158	28.7	26.7	15.5	32.6
50 g/kg Coarse	−	806	1364	334	88.4	79.6	32.6	54.8
50 g/kg Fine	−	557	981	251	93.2	66.8	32.5	34.7
0	+	532	1169	270	21.8	80.9	33.6	36.9
50 g/kg Coarse	+	539	1259	167	61.2	55.5	34.5	36.3
50 g/kg Fine	+	410	1433	237	129	163	29.7	38.2
Pooled SEM		149	379	67.9	23.0	26.6	8.65	10.7
*p*-Value		0.255	0.733	0.209	0.457	0.162	0.761	0.903

STB, stimbiotic; SCFAs, short-chain fatty acids; BCFAs, branched-chain fatty acids. *n* = 16, 24, and 6 for the main effect means of WB, STB, and interaction effect means, respectively. ^a,b^ Means in a column, within a group, with different superscripts are significantly different (*p* < 0.05).

## Data Availability

The original contributions presented in the study are included in the article, further inquiries can be directed to the corresponding author.
